# Relationship Between Quality of Life and Sports Performance Among Athletes with Disabilities: A Focus on Individual Sports

**DOI:** 10.3390/healthcare13222919

**Published:** 2025-11-14

**Authors:** Fatemeh Ahmadi, Mohammad Mehdi Khaleghi, Abdosaleh Zar, Josyula Tejaswi, Karuppasamy Govindasamy, Viorel Petru Ardelean, Vasile Emil Ursu, Vlad Adrian Geantă

**Affiliations:** 1 Department of Sport Science, School of Literature and Humanities, Persian Gulf University, Bushehr 7516913817, Iran; fa_ahmadif@yahoo.com (F.A.); salehzar@gmail.com (A.Z.); 2 Persian Gulf Sports, Nutrition and Wellness Research and Technology Group, Persian Gulf University, Bushehr 7516913817, Iran; khaleghii1379@gmail.com; 3 Department of Exercise Physiology, Faculty of Sport Sciences, Shahid Chamran University of Ahvaz, Ahvaz 6135783151, Iran; 4 Department of Physical Education Pedagogy, Lakshmibai National Institute of Physical Education, Gwalior 474002, India; tejaswi.p0302@gmail.com; 5 Department of Sports, Recreation and Wellness, Symbiosis International (Deemed University), Hyderabad Campus, Hyderabad 509217, India; 6 Department of Physical Education and Sport, Faculty of Physical Education and Sport, Aurel Vlaicu University of Arad, 310330 Arad, Romania; viorel.ardelean@uav.ro (V.P.A.); vlad.geanta@uav.ro (V.A.G.); 7 Department of Physical Education and Sport, Faculty of Law and Social Sciences, University “1 Decembrie 1918” of Alba Iulia, 510009 Alba Iulia, Romania

**Keywords:** life quality, disabled athletes, athletic performance, solo sports, physical disability

## Abstract

**Highlights:**

**What are the main findings?**

- Athletes with physical disabilities reported high levels of overall quality of life, regardless of gender or competition level.
- No significant correlation was found between athletic performance and quality of life across provincial, national, or international levels.

**What are the implications of the main findings?**

- Participation in individual sports may enhance physical and psychological well-being in athletes with physical disabilities.
- Promoting engagement in individual sports could be a valuable strategy for improving overall quality of life among people with disabilities.

**Abstract:**

**Background/Objectives**: Physical activity and sports participation are widely recognized as effective strategies for enhancing quality of life (QoL) in individuals with disabilities. This study aimed to examine the relationship between QoL and athletic performance among male and female athletes with physical disabilities who participate in individual sports. **Methods**: This descriptive–correlational study involved 338 Iranian athletes with physical disabilities competing at various levels of competition. QoL was measured using the SF-36 questionnaire, and sports performance was assessed based on official competition records. Data were analyzed using SPSS v21, applying descriptive statistics and Pearson correlations. **Results**: Both male and female athletes reported high levels of overall QoL. No statistically significant differences were found between genders in terms of physical health, psychological well-being, or total QoL scores (*p* > 0.05). Furthermore, there were no significant correlations between QoL and sports performance at the provincial, national, or international levels (*p* > 0.05). **Conclusions**: The findings indicate that athletes with physical disabilities report relatively high levels of QoL, irrespective of their competitive achievements or medal standings. Although no statistically significant correlations were observed, participation in individual sports may be linked to better physical functioning and psychological resilience. These associations should be interpreted with caution and do not imply causality. Nonetheless, encouraging such participation could be beneficial in supporting various dimensions of health and promoting social inclusion among individuals with disabilities.

## 1. Introduction

Quality of life (QoL) represents an individual’s overall self-assessment or subjective judgment of well-being and life satisfaction, encompassing physical health and functional ability, mental well-being, emotional happiness, satisfaction with interpersonal relationships, and economic and/or occupational status [1]. The World Health Organization’s WHOQOL framework defines QoL as individuals’ perceptions of their position in life in the context of their culture, value systems, goals, expectations, and concerns [2,3]. Health-related quality of life (HRQoL), a subset of QoL, specifically refers to domains influenced by physical and mental health status [4,5]. Among individuals with disabilities, QoL is shaped by multiple interrelated factors, such as the type and extent of the disability, the nature of family relationships and social support, financial security, and the individual’s sense of usefulness or autonomy [6]. Theoretical models such as Deci and Ryan’s Self-Determination Theory further emphasize the role of intrinsic motivation, competence, and relatedness in fostering well-being and life satisfaction. These frameworks provide a more nuanced understanding of QoL beyond clinical or functional outcomes, highlighting the importance of psychological needs and social integration [7,8].

Individuals with physical disabilities often face significant inequalities across various domains of life, which can profoundly impact their physical and psychological health [9], thereby influencing their overall QoL. A study has demonstrated that people with disabilities generally experience a lower QoL compared to the general population [10]. Due to the partial loss of functional abilities, these individuals often rely on assistive devices to maintain independence and carry out daily activities [11]. Such impairments frequently lead to structural inequalities that directly affect their well-being. Among these, access to physical activity and sports represents a notable area of disparity, influenced by several factors including lack of awareness and positive attitudes, insufficient infrastructure, transportation challenges, societal and architectural barriers [12]. Physical activity and sports are fundamental to the prevention of chronic diseases and the maintenance of health, particularly for individuals with mobility limitations. A growing body of evidence highlights the benefits of exercise across diverse populations with neurological disabilities, such as spinal cord injury, stroke, traumatic brain injury, cerebral palsy, multiple sclerosis, and Parkinson’s disease [13]. Participation in both competitive and non-competitive sports offers individuals with physical disabilities a vital pathway to engage in regular physical activity. The concept of sport participation among people with disabilities is not novel. More than half a century ago, Sir Ludwig Guttmann pioneered the development of organized sports for individuals with disabilities, ultimately leading to the establishment of the Paralympic Games [14].

Recent studies underscore the diverse benefits of adapted sport for individuals with disabilities, particularly in promoting well-being, resilience, and social support [15,16]. Participation in sport is consistently linked to greater life satisfaction among individuals with motor impairments compared to non-participants [17,18]. Beyond physical health, sport enhances subjective well-being [19,20], personal growth, and social integration [21]. Resilience—now viewed as a dynamic process of psychological adaptation—has gained prominence in disability sport research, especially in helping athletes cope with adversity and build meaningful social connections [22,23,24,25]. Positive emotions both foster and result from resilience [26,27]. Similarly, social support from coaches, peers, and family is recognized as a key factor in psychological adjustment and athletic development, contributing to both adaptation and long-term growth [28,29,30].

Researchers have consistently recommended the participation of individuals with disabilities in adapted and therapeutic sports as an effective strategy for enhancing their QoL [17]. Traditionally, competitive sporting events for able-bodied athletes have been recognized as powerful tools for stress reduction and psychological well-being [31]. Emerging evidence also suggests that engagement in physical activity among individuals with disabilities may positively influence their overall QoL. Several studies have explored the impact of physical activity on QoL and psychological well-being in populations with motor impairments [32,33,34], with many reporting a significant positive correlation between higher levels of physical activity and improved QoL in these individuals [35,36].

For instance, the study by Yazicioglu et al. demonstrated that individuals with physical disabilities who participated in adapted sports reported significantly higher levels of QoL and life satisfaction compared to non-participants [32]. In a separate study, Perrier et al. applied the Health Action Process Approach and found that athletic identity and motivational constructs significantly predicted sport participation among individuals with acquired physical disabilities, highlighting psychological mechanisms that support engagement [33]. Additionally, a recent systematic review by Diz et al. concluded that physical activity and sport participation were positively associated with multiple domains of QoL and well-being in individuals with intellectual and developmental disabilities [34].

Despite these findings, the role of competitive sports participation—as opposed to general physical activity—in promoting QoL among people with disabilities has received comparatively less attention. Competitive sporting events provide athletes with disabilities unique opportunities not only to showcase their skills but also to enhance their physical health and overall well-being [37]. However, the relationship between sports performance and QoL among disabled athletes engaged in competitive sports remains underexplored. Team and individual sports are two popular styles of physical activity, each of which has different and important effects on physical and mental health. Team sports usually help improve social relationships and increase vitality by creating motivation and a sense of cooperation, while individual sports allow for greater concentration and peace of mind. Based on this, and because of the differences in the nature of these two types of sports, as well as the differences in the physical and mental state affected by these two types of sports, we selected individual athletes.

Therefore, the present study aims to investigate the relationship between QoL and sports performance among veteran and disabled athletes, with a particular focus on individual sports disciplines. This research seeks to better understand how participation in individual sports may influence various dimensions of QoL, including physical, psychological, and social well-being, in this specific athletic population. It is hypothesized that higher levels of sports performance are positively associated with greater QoL among veteran and disabled athletes. Specifically, athletes demonstrating superior performance in individual sports are expected to report higher scores in both physical and psychological components of QoL.

## 2. Materials and Methods

### 2.1. Participants

This descriptive–correlational study was carried out in Iran in 2021. The target population comprised athletes with physical disabilities who competed at the provincial, national, and international levels. From an estimated total of 20,000 individual sports (such as: Shooting (rifle, pistol), Zurkhaneh exercise, Athletics, Cycling, Table tennis, Weightlifting) athletes, a sample of 338 individuals (mean of age: 37.18 ± 11.13 years) was selected using the Morgan sampling table and convenience sampling approach [38]. Eligibility criteria included: voluntary participation with informed consent, absence of any diagnosed mental disorders, and willingness to complete the research questionnaire. Participants who later withdrew or expressed unwillingness to continue were excluded from the study. Ethical approval was obtained from the Jahrom University of Medical Sciences Ethics Committee (Fars Province, Iran) under the reference number IR.JUMS.REC.1399.045. All procedures were conducted in accordance with ethical standards, ensuring the confidentiality and anonymity of participant data.

### 2.2. Procedure

Following initial approval from the Federation of Veterans and People with Disabilities, formal coordination was established with the relevant provincial sports delegations. All procedural details and documentation were distributed to the provincial offices via official correspondence from the federation. Prior to data collection, participants were provided with a clear explanation of the study objectives and procedures, particularly regarding the assessment of QoL. Informed consent was obtained, and participants subsequently completed the standardized QoL questionnaire [39]. Athletic performance was determined using official records from the provincial delegations and the Federation, which documented the athletes’ competitive level (i.e., provincial, national, or international). These competition levels were used as an indicator of each athlete’s sport performance status.

### 2.3. Quality of Life Assessment Tool

To evaluate participants’ QoL, the 36-Item Short Form Survey (SF-36) developed by Ware and Sherbourne (1992) [39] was employed. This standardized self-report instrument assesses multiple dimensions of health-related QoL, encompassing eight distinct domains: Social Function, Vitality, Mental Health, Emotional problems, Body Pain, Understanding of your life, Physical Function, and Physical health [39]. Each domain is scored on a scale ranging from 0 to 100, with higher scores indicating more favorable outcomes.

The psychometric properties of the SF-36 have been validated in various populations, including Iranian cohorts. Previous studies have reported acceptable internal consistency coefficients for the eight domains, ranging from 0.70 to 0.85, with test–retest reliability coefficients between 0.43 and 0.79 across a one-week interval [40,41]. Furthermore, Montazeri et al. (2009) confirmed the questionnaire’s validity and reliability within the Iranian context, reporting Cronbach’s alpha values of 0.73 and 0.72 for the 12-item physical functioning component [42]. Retest analyses also indicated satisfactory reliability, with Cronbach’s alpha coefficients of 0.89 and 0.76 for physical and psychological dimensions, respectively [43]. In the current study, the internal consistency of the SF-36 was assessed using Cronbach’s alpha, which yielded a reliability coefficient of 0.80.

### 2.4. Evaluation of Athletic Performance

Athletic performance was assessed based on the official rankings and competition outcomes achieved by each participant. These data were obtained through coordination with provincial sports authorities and the Federation for Veterans and Persons with Disabilities, ensuring accurate classification of athletes’ competitive levels (provincial, national, or international).

### 2.5. Statistical Analysis

All data were analyzed using SPSS software version 21 (IBM Corp., Armonk, NY, USA). Descriptive statistics, including means and standard deviations, were calculated for all relevant variables. To assess the associations between study variables, Pearson’s product–moment correlation coefficient was employed. Multivariate regression was used to analyze the relationship between variables. In cases where the assumption of normality was violated, the non-parametric Mann–Whitney U test was applied to compare outcomes between male and female participants. A significance level of *p* < 0.05 was considered statistically meaningful.

## 3. Results

### 3.1. Quality of Life Among Individual Sport Athletes

**Figure 1:**
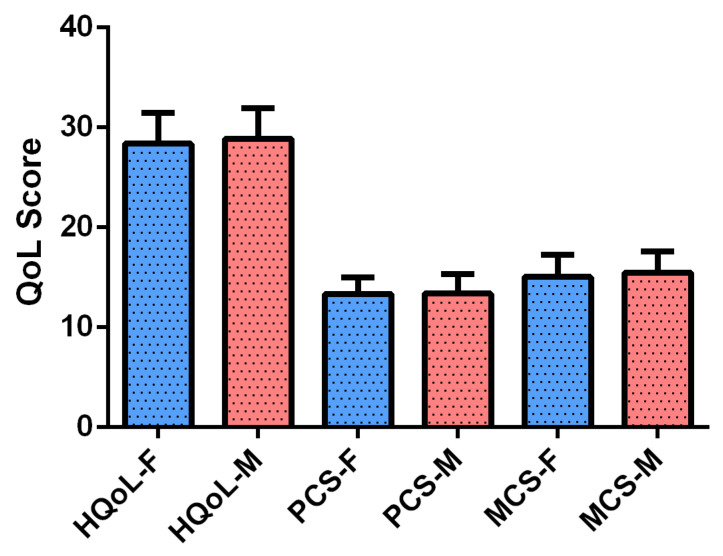
Quality of Life Scores among Male and Female Athletes in Individual Sports. Data are presented as the mean ± standard error of the mean. HQol-F: health-related quality of life-Female; HQol-M: health-related quality of life-Male; MCS-F: Mental Component Summary-Female; MCS-M: Mental Component Summary-Male; PCS-F: physical component summary-Female; PCS-M: physical component summary-Male.

The results of the Mann–Whitney U test comparing QoL between male and female athletes in individual sports (Table 1, Figure 1) revealed no statistically significant differences in overall mental health, physical health, or total QoL scores between the two groups (*p* > 0.05).

The descriptive analysis of QoL scores among individual sport athletes revealed comparable mean values between men and women across most domains. The Mental Component Summary was slightly higher in women (15.47 ± 2.11) than in men (15.03 ± 2.34), while the Physical Component Summary showed nearly identical scores (13.38 ± 1.91 for women vs. 13.35 ± 1.69 for men). Vitality scores were equal (3.97 ± 1.20 for men and 3.97 ± 1.16 for women), and similar patterns were observed in Mental Health (6.9 ± 1.52 for men and 6.35 ± 1.27 for women), Emotional problems (2.50 ± 0.78 for men and 2.62 ± 0.87 for women), Body Pain (3.88 ± 1.23 for men and 3.85 ± 1.19 for women), Physical Function (3.66 ± 1.37 for men and 3.47 ± 1.39 for women), and Physical Health (12.48 ± 0.82 for men and 14.46 ± 0.80 for women). Notably, women reported significantly higher scores in Social Function (2.52 ± 1.36 vs. 2.16 ± 1.25; *p* = 0.016) and Understanding of Life (3.58 ± 1.18 vs. 3.32 ± 1.06; *p* = 0.027). Overall, the HRQoL score was slightly higher in women (28.86 ± 3.04) than in men (28.39 ± 3.05), though this difference was not statistically significant.

### 3.2. Analysis of the Number of Championships Among Individual Sport Athletes

The Mann–Whitney U test was applied to compare the number of championships between female and male athletes in individual sports (Table 2). The results revealed that female athletes had significantly higher numbers of championships at all competitive levels compared to their male counterparts. Specifically, the mean number of provincial-level championships among females was 2.21 ± 0.90, compared to 2.19 ± 0.98 in males; national-level championships were 3.43 ± 1.33 in females versus 3.10 ± 1.47 in males; and international-level championships were 3.80 ± 1.24 in females compared to 3.44 ± 1.48 in males. These differences were statistically significant (*p* < 0.05).

### 3.3. Association Between Quality of Life and Athletic Performance Across Competitive Levels

The results of correlation analysis indicated no statistically significant relationship between QoL and athletic performance among female individual sport athletes at the provincial level (r = −0.102, *p* = 0.226), national level (r = −0.089, *p* = 0.291), or international level (r = −0.065, *p* = 0.443). Similarly, no significant association was observed between QoL and athletic performance among male athletes at the provincial (r = 0.051, *p* = 0.479), national (r = −0.010, *p* = 0.893), or international levels (r = 0.060, *p* = 0.401). When data from male and female athletes were analyzed collectively, the results also revealed no significant correlation between QoL and athletic performance at the provincial (r = −0.018, *p* = 0.738), national (r = −0.049, *p* = 0.372), or international levels (r = 0.002, *p* = 0.977) (Table 3).

### 3.4. Association Between Physical Health (As a Domain of Quality of Life) and Athletic Performance Across Competitive Levels

The results revealed no statistically significant association between the physical health component of QoL and athletic performance among female athletes in individual sports at the provincial level (r = −0.044, *p* = 0.607), national level (r = 0.022, *p* = 0.796), or international level (r = −0.052, *p* = 0.539). Similarly, no meaningful correlations were found between physical health scores and athletic championships in male athletes at the provincial (r = −0.036, *p* = 0.618), national (r = −0.056, *p* = 0.432), or international levels (r = −0.035, *p* = 0.631). When data from both sexes were combined, the analysis also showed no statistically significant relationship between physical health and athletic performance at the provincial (r = −0.039, *p* = 0.469), national (r = −0.030, *p* = 0.583), or international levels (r = −0.041, *p* = 0.450) (Table 4).

### 3.5. Association Between Mental Health (As a Component of Quality of Life) and Athletic Performance at Provincial, National, and International Levels

The findings indicated no statistically significant correlation between the mental health domain of QoL and athletic performance among female athletes in individual sports at the provincial level (r = −0.102, *p* = 0.228), national level (r = −0.132, *p* = 0.116), or international level (r = −0.047, *p* = 0.578). For male athletes, a significant association was observed between mental health and athletic performance at the international level (r = −0.019, *p* = 0.787), although this correlation was not statistically significant. In contrast, no significant relationship was found at the provincial (r = −0.007, *p* = 0.919) or national levels (r = 0.146, *p* = 0.042), although the *p*-value at the national level suggests a borderline result. When data from both sexes were combined, no statistically significant correlations were observed between mental health and athletic performance at the provincial (r = 0.007, *p* = 0.894), national (r = −0.042, *p* = 0.436), or international levels (r = 0.036, *p* = 0.509) (Table 5).

### 3.6. Multivariate Regression Study on the Relationship Between Quality of Life Components and Sports Performance

The ANOVA results show that there is a significant difference between the variables (*p* = 0.008). Multiple regression results show that the components of physical performance (Standardized Coefficients = 0.210, *p* = 0.023) and overall perception (Standardized Coefficients =−0.318, *p* = 0.001), respectively, have the greatest relationship with the sports performance of female athletes at the provincial level.

The ANOVA results show that there is a significant difference between the variables (*p* = 0.029). Multiple regression results show that the components of physical performance (Standardized Coefficients = 0.223, *p* = 0.013) and overall perception (Standardized Coefficients = −0.243, *p* = 0.012), respectively, have the greatest relationship with the sports performance of female athletes at the National level.

The ANOVA results show that there is a significant difference between the variables (*p* = 0.001). Multiple regression results show that the components of Social Function (Standardized Coefficients = 0.611, *p* = 0.001), Vitality (Standardized Coefficients = 0.606, *p* = 0.001), Body Pain (Standardized Coefficients = 0.449, *p* = 0.001), Mental Health (Standardized Coefficients = −0.212, *p* = 0.005), Emotional problems (Standardized Coefficients = −0.249, *p* = 0.019), Understanding of your life (Standardized Coefficients = −0.367, *p* = 0.001), respectively, have the greatest relationship with the sports performance of female athletes at the International level.

The ANOVA results show that there is a significant difference between the variables (*p* = 0.001). Multiple regression results show that the component of Emotional problems (Standardized Coefficients = 0.288, *p* = 0.001), have the greatest relationship with the sports performance of male athletes at the provincial level.

The ANOVA results show that there is a significant difference between the variables (*p* = 0.005). Multiple regression results show that there was no difference in the various components of male athletes’ athletic performance at the provincial level.

The ANOVA results show that there is not a significant difference between the variables (*p* = 0.145). Multiple regression results show that the components of Emotional problems (Standardized Coefficients = 0.228, *p* = 0.012) and Mental Health (Standardized Coefficients = 0.154, *p* = 0.042) have the greatest relationship with the sports performance of male athletes at the international level.

## 4. Discussion

The results indicated that, overall, both male and female athletes engaged in competitive sports exhibited high levels of QoL. Notably, no statistically significant differences were observed between male and female participants in terms of overall QoL, general physical health, or general mental health scores. Nevertheless, small yet statistically significant differences were observed in the domains of Social Function and Understanding of Life. These findings underscore the potentially transformative role of competitive sports participation in enhancing the QoL among individuals with disabilities—regardless of gender. Though, gender may serve as a contributing factor in the emergence of individual differences within the subcomponents of the Mental and Physical domains, which in turn may influence overall sleep quality. These results also confirm the study’s hypothesis that higher levels of sports performance are positively associated with greater QoL among veteran and disabled athletes, particularly in individual sports disciplines. This suggests that engagement in structured athletic activities may serve as a critical factor in promoting physical and psychological well-being in this population.

It appears that the nature of sport and physical activity contributes to the satisfaction of internal emotional needs through increased sociability, a greater openness to new experiences and learned techniques, and enhanced motivation for social engagement, such as participation in competitive sports [44]. These elements may directly influence improvements in QoL. Through mechanisms such as fostering self-efficacy, boosting self-confidence, and reducing stress levels, sport and physical activity have been shown to improve psychological components of well-being, thereby promoting mental health and, in turn, enhancing overall QoL among individuals with disabilities [45]. Although team sports are often associated with improved psychological outcomes due to social interactions and group cohesion, the high QoL reported by participants in individual sports in this study suggests that physical activity, regardless of its type, can lead to the development of both physical and psychological attributes that enhance life satisfaction. For athletes with disabilities, achieving success and winning medals in competitive individual sports may contribute to enhanced perceptions of QoL; however, given the nonsignificant statistical findings, this interpretation should be approached with caution and considered exploratory rather than conclusive. Supporting this notion, a previous study conducted on professional disabled cyclists reported high levels of QoL among participants [46]. Another study revealed that wheelchair basketball athletes from seven men’s and three women’s national teams experienced notable physical, psychological, and social benefits, including improved functional health, enhanced self-belief, and stronger social connections [47].

In the present study, no statistically significant relationship was observed between overall QoL and athletic performance, as measured by the number of medals earned at the provincial, national, and international levels. Additionally, no significant differences were found in physical and mental health dimensions of QoL among male and female para-athletes across these competitive levels. Although similar findings have been reported in other studies where QoL was not significantly associated with athletic performance based on medal count, research by Ciampolini et al. (2017) indicated that professional disabled tennis players reported a high QoL [37]. Comparable results were also observed among disabled basketball athletes [9]. It is widely recognized that sport and physical activity positively influence physical and mental health, as well as overall QoL among individuals with disabilities. Previous research has shown that a higher QoL is strongly associated with greater life satisfaction [32], which in turn enables individuals with disabilities to maintain better control over their lives, sustain healthier social relationships, and exhibit higher commitment to activities that contribute to personal or societal advancement [37]. A study of male wheelchair handball athletes found that various sociodemographic and sport-related factors—such as marital status, origin of disability, level of independence, training frequency, and use of custom-made wheelchairs—significantly influence performance and QoL [48].

While the current study did not find a statistically significant association between athletic performance and scores of overall QoL, physical health, or mental well-being, both male and female participants involved in individual competitive sports reported high—and in many cases, excellent—levels of QoL. Although competitive environments are often assumed to exert stress that might hinder athletic performance or diminish well-being, these findings suggest that participation in competitive sport may, in fact, be associated with favorable perceptions of life quality among disabled athletes. This observation may serve as a motivational factor not only for the athletes themselves but also for their families and support networks, encouraging further engagement in competitive sports.

Grounded in self-determination theory and the biopsychosocial model of disability, sport participation has been shown to promote core determinants of QoL, including autonomy, social inclusion, and physical competence [49]. These factors collectively contribute to improved psychological and social outcomes such as enhanced self-confidence, overall well-being, and a stronger sense of personal accomplishment [49,50]. For instance, one study highlighted the role of school curricula in fostering early interest and engagement in sport among individuals with physical impairments, thereby facilitating positive initial experiences. Such educational initiatives may help reduce negative stereotypes and promote long-term social inclusion [50]. Notably, these findings underscore that self-perceived achievement may exert a greater influence on QoL than objective measures of athletic performance.

The lack of a statistically significant relationship between QoL and athletic performance observed in this study appears to be justifiable, given that the participating para-athletes exhibited high QoL scores regardless of their medal achievements. This suggests that their perceived well-being may not be directly contingent upon competitive success. Previous research indicates that participation in national and international sporting events enables athletes with disabilities to engage with peers from diverse backgrounds and cultural contexts, thereby fostering broader social interactions and enhancing their psychosocial well-being [51]. This increased social exposure and engagement can play a pivotal role in improving mental health, which in turn may contribute to an elevated QoL—independent of athletic performance metrics such as medal counts. These findings underscore the critical importance of encouraging active participation in sports and competitive events among individuals with disabilities. Beyond the potential for athletic accomplishment, involvement in sports serves as a powerful mechanism for promoting both physical and psychological health, ultimately leading to improvements in overall QoL for this population. Promoting access to competitive sports, therefore, may represent a valuable strategy in broader efforts to support the well-being and social inclusion of individuals with disabilities.

Additionally, in the present study, the absence of a statistically significant association between QoL and sports performance may be attributed to several factors. First, the “number of championships” used as an indirect indicator of performance may not accurately capture the multifaceted nature of athletic success, which often involves technical, tactical, and psychological dimensions beyond competitive outcomes. Objective performance metrics such as times, distances, or scores could provide a more precise reflection of individual capabilities. However, due to data unavailability across different sports disciplines, the number of championships was used as a standardized and comparable proxy measure.

Furthermore, QoL is influenced by a complex interplay of physical, psychological, social, and environmental factors that extend beyond athletic performance. It is possible that non-sport-related determinants—such as access to rehabilitation services, social support, financial security, or perceived stigma—played a stronger role in shaping QoL among the participating athletes. Therefore, the lack of a significant relationship may reflect the broader, multidimensional nature of QoL rather than the absence of interaction between sport participation and well-being.

Finally, the similar QoL scores between male and female athletes might suggest that participation in sports, regardless of gender or performance level, provides comparable psychosocial benefits, reinforcing the inclusive and empowering role of sports participation among individuals with disabilities.

### Limitations, Practical Implications, and Future Research Directions

This study has several limitations that should be acknowledged. First, the use of a convenience sampling method may limit the generalizability of the findings due to potential selection bias. Participants who chose to take part in the study may differ systematically from those who did not, particularly in terms of motivation, access to resources, or prior experiences with sports. Second, the cross-sectional design precludes any inference of causality between sports participation and QoL outcomes. Third, the reliance on self-reported data, particularly for QoL assessment, introduces the possibility of response bias, including social desirability and recall inaccuracies. Fourth, the performance metric used—categorizing athletes by provincial, national, or international competition level—serves as a coarse ordinal proxy and does not capture the nuances of actual athletic performance. While this classification was chosen to facilitate comparison across competitive tiers, it may oversimplify the complexity of athletic achievement and training intensity. Fifth, the absence of control variables such as age, years of experience, and type of disability limits the interpretability of observed correlations. These factors likely influence both sports participation and QoL, and their omission may confound the results. Sixth, the study did not incorporate detailed classifications of disability (e.g., type, duration, or etiology), which could have provided more granular insights into how different disability profiles interact with sports engagement and psychosocial outcomes. These methodological constraints should be considered when interpreting the results.

Despite these limitations, the findings offer valuable insights for practitioners, policymakers, and researchers. Rehabilitation specialists, sports psychologists, and disability advocates may find the results useful in promoting inclusive sports programs. The observed high levels of QoL among athletes with disabilities—regardless of competitive success—highlight the potential psychosocial benefits of regular participation in individual sports. These findings may inform the development of community-based initiatives and national policies aimed at increasing access to adaptive sports for individuals with physical disabilities.

To build on the current findings, future studies should consider employing longitudinal or experimental designs to better assess causal relationships between sports participation and QoL. Intervention-based research could also explore the specific mechanisms through which physical activity influences psychological and social well-being in this population. Additionally, incorporating qualitative methods may provide deeper insights into the lived experiences of athletes with disabilities, enriching our understanding of the contextual factors that shape their QoL. Future research should also include relevant control variables and detailed disability classifications to enhance the precision and applicability of findings.

## 5. Conclusions

The findings of this study indicate that both male and female athletes with disabilities who engage in competitive sports report relatively high levels of quality of life. While no statistically significant correlations were found between athletic performance and quality of life, the data suggest that participation in sports—irrespective of medal achievements—may be associated with enhanced physical and psychological well-being. These associations should be interpreted cautiously and not as evidence of causality.

Based on these observations, a practical recommendation is to encourage individuals with disabilities, their families, and relevant stakeholders to support regular training and involvement in competitive sports, as such engagement may contribute to improved health and social integration. Future research employing longitudinal or intervention-based designs is warranted to better understand the potential causal pathways and long-term effects of sports participation on quality of life in this population.

## Figures and Tables

**Table 1:** The results of the Mann–Whitney U test on quality of life among individual sport athletes.

Variable	Men	Women	*p* Value
**Mental Component Summary**	15.03 ± 2.34	15.47 ± 2.11	0.079
Social Function	2.16 ± 1.25	2.52 ± 1.36	0.016 *
Vitality	3.97 ± 1.20	3.97 ± 1.16	0.953
Mental Health	6.9 ± 1.52	6.35 ± 1.27	0.813
Emotional problems	2.50 ± 0.78	2.62 ± 0.87	0.206
**Physical component summary**	13.35 ± 1.69	13.38 ± 1.91	0.857
Body Pain	3.88 ± 1.23	3.85 ± 1.19	0.905
Understanding of your life	3.32 ± 1.06	3.58 ± 1.18	0.027 *
Physical Function	3.66 ± 1.37	3.47 ± 1.39	0.199
Physical health	12.48 ± 0.82	14.46 ± 0.80	0.793
**Health-related quality of life**	28.39 ± 3.05	28.86 ± 3.04	0.162

Data are presented as mean ± standard deviation. * Significant difference between female and male groups.

**Table 2:** The results of the Mann–Whitney U test comparing the number of championships in individual sports by gender.

Variable	Female Group	Male Group	Overall Score	*p*
Provincial-level championships	2.21 ± 090	2.19 ± 0.98	2.20 ± 0.33	0.030 *
National-level championships	3.43 ± 1.33	3.10 ± 1.47	3.24 ± 1.42	0.020 *
International-level championships	3.80 ± 1.24	3.44 ± 1.48	3.59 ± 1.40	0.001 *

Data are presented as mean ± standard deviation. * Significant difference between female and male groups.

**Table 3:** The correlation between quality of life and athletic performance at the provincial, national, and international levels among individual sport athletes.

Variable	Group	Sports Performance					
		Provincial Level		National Level		International Level	
		r	*p*	r	*p*	r	*p*
Quality of life	Women	−0.102	0.226	−0.089	0.291	−0.065	0.443
	Men	0.051	0.479	−0.010	0.893	0.060	0.401
	Women + men	−0.018	0.738	−0.049	0.372	0.002	0.977

**Table 4:** The correlation between the physical health domain of quality of life and athletic performance at the provincial, national, and international levels among individual sport athletes.

Variable	Group	Sports Performance					
		Provincial Level		National Level		International Level	
		r	*p*	r	*p*	r	*p*
Physical health	Women	−0.044	0.607	−0.022	0.796	−0.052	0.539
	Men	−0.036	0.618	−0.056	0.432	−0.035	0.631
	Women + men	−0.039	0.469	−0.030	0.583	−0.041	0.450

**Table 5:** A correlation analysis between the mental health dimension of quality of life and athletic performance across different competitive levels in individual sports athletes.

Variable	Group	Sports Performance					
		Provincial Level		National Level		International Level	
		r	*p*	r	*p*	r	*p*
Mental health	Women	−0.102	0.228	−0.132	0.116	−0.047	0.578
	Men	−0.019	0.787	−0.007	0.919	0.146	0.042 *
	Women + men	0.007	0.894	−0.042	0.436	0.036	0.509

* Statistically significant correlation (*p* < 0.05).

## Data Availability

The data that support the findings of this study are available from the corresponding author upon reasonable request. Data access is restricted to protect participant confidentiality, as the dataset contains sensitive human-related information. However, anonymized or summarized data can be shared upon reasonable request to ensure transparency while maintaining ethical standards.

## Abbreviations

The following abbreviations are used in this manuscript:

QoL	Quality of life
HRQoL	Health-related quality of life
SF-36	36-Item Short Form Survey
HQol-F	Health-related quality of life-Female
HQol-M	Health-related quality of life-Male
MCS-F	Mental Component Summary-Female
MCS-M	Mental Component Summary-Male
PCS-F	Physical component summary-Female
PCS-M	physical component summary-Male
PLA-F	Provincial-level championships-Female
PLA-M	Provincial-level championships-Male
NLA-F	National-level championships-Female
NLA-M	National-level championships-Male
ILA-F	International-level championships-Female
ILA-M	International-level championships-Male

## References

[1] Bjornson K.F., McLaughlin J.F. (2001). The measurement of health-related quality of life (HRQL) in children with cerebral palsy. Eur. J. Neurol..

[2] Group W. (1994). Development of the WHOQOL: Rationale and current status. Int. J. Ment. Health.

[3] Kim S. (2024). World Health Organization quality of life (WHOQOL) assessment. ENCYCLOPEDIA of Quality of Life and Well-Being Research.

[4] Kim J., Chung H., Amtmann D., Salem R., Park R., Askew R.L. (2014). Symptoms and quality of life indicators among children with chronic medical conditions. Disabil. Health J..

[5] Enssle F., Kabisch N. (2020). Urban green spaces for the social interaction, health and well-being of older people—An integrated view of urban ecosystem services and socio-environmental justice. Environ. Sci. Policy.

[6] Kamelska A.M., Mazurek K. (2015). The assessment of the quality of life in visually impaired people with different level of physical activity. Phys. Cult. Sport.

[7] Deci E.L., Ryan R.M. (2000). The “what” and “why” of goal pursuits: Human needs and the self-determination of behavior. Psychol. Inq..

[8] Ryan R.M., Deci E.L. (2020). Intrinsic and extrinsic motivation from a self-determination theory perspective: Definitions, theory, practices, and future directions. Contemp. Educ. Psychol..

[9] Chatzilelecas E., Filipovic B., Petrinovic L. (2015). Differences in quality of life according to the level of physical activity between two groups of basketball players in the wheelchairs. SportLogia.

[10] Tahmasebi H., Abasi E., Zafari M., Darvish Gkezri H. (2016). Comparison of the quality of life of paraplegic veterans and disables; Case study of Mazandaran Province, Iran. Iran. J. War Public Health.

[11] Borade N., Ingle A., Nagarkar A. (2021). Lived experiences of people with mobility-related disability using assistive devices. Disabil. Rehabil. Assist. Technol..

[12] Koo K.M., Kim C.J., Park C.H., Byeun J.K., Seo G.W. (2016). Restrictions of physical activity participation in older adults with disability: Employing keyword network analysis. J. Exerc. Rehabil..

[13] Laskowski E.R., Lexell J. (2012). Exercise and sports for health promotion, disease, and disability. PM&R.

[14] Sahlin K.B., Lexell J. (2015). Impact of Organized Sports on Activity, Participation, and Quality of Life in People with Neurologic Disabilities. PM&R.

[15] Caddick N., Smith B. (2014). The impact of sport and physical activity on the well-being of combat veterans: A systematic review. Psychol. Sport Exerc..

[16] Hogan C.L., Catalino L.I., Mata J., Fredrickson B.L. (2015). Beyond emotional benefits: Physical activity and sedentary behaviour affect psychosocial resources through emotions. Psychol. Health.

[17] Blauwet C., Willick S.E. (2012). The Paralympic Movement: Using sports to promote health, disability rights, and social integration for athletes with disabilities. PM&R.

[18] Frank C., Land W.M., Schack T. (2013). Mental representation and learning: The influence of practice on the development of mental representation structure in complex action. Psychol. Sport Exerc..

[19] Ku P.-W., Fox K.R., Chang C.-Y., Sun W.-J., Chen L.-J. (2014). Cross-Sectional and Longitudinal Associations of Categories of Physical Activities with Dimensions of Subjective Well-Being in Taiwanese Older Adults. Soc. Indic. Res..

[20] Olsson L.A., Hurtig-Wennlöf A., Nilsson T.K. (2014). Subjective well-being in Swedish active seniors and its relationship with physical activity and commonly available biomarkers. Clin. Interv. Aging.

[21] Mira T., Costa A.M., Jacinto M., Diz S., Monteiro D., Rodrigues F., Matos R., Antunes R. (2023). Well-Being, Resilience and Social Support of Athletes with Disabilities: A Systematic Review. Behav. Sci..

[22] Nicholls A.R., Morley D., Perry J.L. (2016). The model of motivational dynamics in sport: Resistance to peer influence, behavioral engagement and disaffection, dispositional coping, and resilience. Front. Psychol..

[23] Lu F.J.H., Lee W.P., Chang Y.-K., Chou C.-C., Hsu Y.-W., Lin J.-H., Gill D.L. (2016). Interaction of athletes’ resilience and coaches’ social support on the stress-burnout relationship: A conjunctive moderation perspective. Psychol. Sport Exerc..

[24] Ong A.D., Bergeman C.S., Boker S.M. (2009). Resilience comes of age: Defining features in later adulthood. J. Pers..

[25] Fletcher D., Sarkar M. (2012). A grounded theory of psychological resilience in Olympic champions. Psychol. Sport Exerc..

[26] Cohn M.A., Fredrickson B.L., Brown S.L., Mikels J.A., Conway A.M. (2009). Happiness unpacked: Positive emotions increase life satisfaction by building resilience. Emotion.

[27] Ryff C.D. (2014). Self Realization and Meaning Making in the Face of Adversity: A Eudaimonic Approach to Human Resilience. J. Psychol. Afr..

[28] Feeney B.C., Collins N.L. (2015). A new look at social support: A theoretical perspective on thriving through relationships. Personal. Soc. Psychol. Rev..

[29] Ascione A., Belfiore P., Di Palma D. (2018). Sports program to promote the wellbeing of people with disabilities. Acta Medica Mediterr..

[30] Sheridan D., Coffee P., Lavallee D. (2014). A systematic review of social support in youth sport. Int. Rev. Sport Exerc. Psychol..

[31] Guo C., Chen F., Wang Y. (2025). The Psychological Impact of Competitive Sports Participation on Adolescent Athletes: An Analysis of Coping Mechanisms and Performance Outcomes. J. Ration.–Emotive Cogn.–Behav. Ther..

[32] Yazicioglu K., Yavuz F., Goktepe A.S., Tan A.K. (2012). Influence of adapted sports on quality of life and life satisfaction in sport participants and non-sport participants with physical disabilities. Disabil. Health J..

[33] Perrier M.-J., Sweet S.N., Strachan S.M., Latimer-Cheung A.E. (2012). I act, therefore I am: Athletic identity and the health action process approach predict sport participation among individuals with acquired physical disabilities. Psychol. Sport Exerc..

[34] Diz S., Jacinto M., Costa A.M., Monteiro D., Matos R., Antunes R. (2024). Physical activity, quality of live and well-being in individuals with intellectual and developmental disability. Healthcare.

[35] Ebrahimi A., Kazemi A., Ebrahimi A. (2016). Wheelchair design and its influence on physical activity and quality of life among disabled individuals. Iran. Rehabil. J..

[36] Ponzano M., Buren R., Adams N.T., Jun J., Jetha A., Mack D.E., Ginis K.A.M. (2024). Effect of Exercise on Mental Health and Health-related Quality of Life in Adults with Spinal Cord Injury: A Systematic Review and Meta-analysis. Arch. Phys. Med. Rehabil..

[37] Ciampolini V., Columna L., Lapolli B., Iha T., Grosso E.C., Silva D.A.S., Galatti L.R. (2017). Quality of life of Brazilian wheelchair tennis athletes across competitive and elite levels. Mot. Rev. Educ. Física.

[38] Krejcie R.V., Morgan D.W. (1970). Determining sample size for research activities. Educ. Psychol. Meas..

[39] Ware J.E., Sherbourne C.D. (1992). The MOS 36-item short-form health survey (SF-36): I. Conceptual framework and item selection. Med. Care.

[40] Asghari Moghaddam M., Faghehi S. (2003). Validity of the sf-36 health survey questionnaire in two iranian samples. Clin. Psychol. Personal..

[41] Montazeri A., Goshtasebi A., Vahdaninia M., Gandek B. (2005). The Short Form Health Survey (SF-36): Translation and validation study of the Iranian version. Qual. Life Res..

[42] Montazeri A., Vahdaninia M., Mousavi S.J., Omidvari S. (2009). The Iranian version of 12-item Short Form Health Survey (SF-12): Factor structure, internal consistency and construct validity. BMC Public Health.

[43] Ware J.E., Kosinski M., Keller S.D. (1996). A 12-Item Short-Form Health Survey: Construction of scales and preliminary tests of reliability and validity. Med. Care.

[44] Zar A., Reza S.H., Ahmadi F., Nikolaidis P.T., Safari M.A., Keshazarz M.H., Ramsbottom R. (2022). Investigating the Relationship between Big Five Personality Traits and Sports Performance among Disabled Athletes. BioMed Res. Int..

[45] Mason O.J., Holt R. (2012). Mental health and physical activity interventions: A review of the qualitative literature. J. Ment. Health.

[46] Ahmadi M.A., Zar A., Vahdatpoor H., Ahmadi F. (2018). The role of professional sports on quality of sleep and life in veterans and disabled professional cycling team athletes. Health Dev. J..

[47] Kirk T., McKay C., Holland K. (2025). “A Kind of Therapy”: Wheelchair Sport Athletes and Health-Related Quality of Life. Res. Q. Exerc. Sport.

[48] Calheiros D.d.S., Neto J.L.C., Melo F.A.P.d., Pedrosa de Melo F.Í., Munster M.d.A.v. (2021). Quality of life and associated factors among male wheelchair handball athletes. Percept. Mot. Ski..

[49] Ryan R.M., Deci E.L. (2000). Self-determination theory and the facilitation of intrinsic motivation, social development, and well-being. Am. Psychol..

[50] Ballas J., Buultjens M., Murphy G., Jackson M. (2022). Elite-level athletes with physical impairments: Barriers and facilitators to sport participation. Disabil. Soc..

[51] Unver S., Tulin A., Cavusoglu G., Vedat E., Yamak B. (2015). A comparison of levels of quality of life, depression and loneliness among athletes with different levels of training. Educ. Res. Rev..

